# *Toxocara* infection: seroprevalence and associated risk factors among primary school children in central China

**DOI:** 10.1051/parasite/2020028

**Published:** 2020-05-05

**Authors:** Shuai Wang, Haoran Li, Zhijun Yao, Pengju Li, Dong Wang, Haizhu Zhang, Qing Xie, Zhenchao Zhang, Xiangrui Li

**Affiliations:** 1 Xinxiang Key Laboratory of Pathogenic Biology, School of Basic Medical Sciences, Xinxiang Medical University Xinxiang 453003 Henan PR China; 2 MOE Joint International Research Laboratory of Animal Health and Food Safety, College of Veterinary Medicine, Nanjing Agricultural University Nanjing 210095 Jiangsu PR China

**Keywords:** *Toxocara*, Primary school children, Seroprevalence, Risk factors, Central China

## Abstract

Toxocariasis is a zoonotic disease that poses a threat to public health worldwide. In the present study, we investigated the seroprevalence of *Toxocara* infection among primary school children in Henan province, central China, which was previously unknown. Sera from 2451 primary school children were collected from September 2015 to October 2018, and evaluated for anti-*Toxocara* antibodies by enzyme-linked immunosorbent assay (ELISA). The overall seroprevalence of *Toxocara* infection was 5.14% (126/2451). The main risk factors related to *Toxocara* infection identified in this study were the age of children, residence area of children, contact with cats or dogs, and exposure to soil. Hand washing before eating was considered to be a protective factor. These findings demonstrate that *Toxocara* infection is relatively common among primary school children in Henan province.

## Abstract (fr)

La toxocarose est une zoonose qui représente une menace pour la santé publique dans le monde. Dans la présente étude, nous avons étudié la séroprévalence de l’infection à *Toxocara* chez les enfants des écoles primaires de la province du Henan, en Chine centrale, qui était auparavant inconnue. Des sérums de 2451 enfants du primaire ont été collectés de septembre 2015 à octobre 2018 et évalués pour les anticorps anti-*Toxocara* par dosage immuno-enzymatique (ELISA). La séroprévalence globale de l’infection à *Toxocara* était de 5,14 % (126/2451). Les principaux facteurs de risque liés à l’infection à *Toxocara* identifiés dans cette étude étaient l’âge des enfants, la zone de résidence des enfants, le contact avec des chats ou des chiens et l’exposition au sol. Le lavage des mains avant de manger a été considéré comme un facteur de protection. Ces résultats démontrent que l’infection à *Toxocara* est relativement courante chez les enfants des écoles primaires de la province du Henan.

## Introduction

Toxocariasis is a worldwide zoonotic infection caused by the ascarid larvae of the *Toxocara* genus, including *Toxocara canis* (*T. canis*) and *Toxocara cati* (*T. cati*). *Toxocara canis* acts as the most frequent cause of toxocariasis, whereas *T. cati* is less common [16]. Their definitive hosts are domestic dogs and cats, respectively. Humans are accidental hosts who become infected by ingesting infective eggs or undercooked meat/viscera of infected paratenic hosts. After ingestion, the eggs hatch and larvae migrate through the intestine and can be carried to multiple organs (heart, liver, lungs, muscle, brain, and eyes) via the bloodstream, causing local reactions and mechanical damage that causes clinical toxocariasis [28]. Infection in humans leads to various disorders accompanied by relevant manifestations. There are four commonly described disorders: convert toxocariasis (CT), neurotoxocariasis (NT) (e.g., eosinophilic meningoencephalitis), ocular larva migrans (OLM), and visceral larva migrans (VLM) [21, 28].

Children usually become infected by accidentally ingesting embryonated eggs of *T. canis* or *T. cati* from the contaminated environment (e.g., soil and water) [24, 27] and dirty hands or, occasionally, by eating invertebrates, such as earthworms [4]. Consumption of undercooked meat from paratenic hosts that contain encapsulated larvae can also result in toxocariasis [14, 34].

In China, *T. canis* and *T. cati* have been widely detected in dogs and cats, respectively [9, 32, 36]. An increasing number of clinical cases of toxocariasis have been reported in children (Table 1; [18, 20, 33, 37–39]). However, little is known about the seroprevalence of *Toxocara* infection among children in China (Table 2; [5, 6, 19, 20]). Most surveys were published in local journals in Chinese, and are not readily accessible to international readers. Furthermore, reports on *Toxocara* seroprevalence among students in primary school in Henan province, central China were still lacking. Consequently, the aim of the current study was to investigate *Toxocara* seroprevalence and relevant risk factors among students in primary school in Henan province.

**Table 1:** Reported clinical cases of toxocariasis in children in the People’s Republic of China.

Provinces/cities	Year	No. of cases	Reference
Chengdu, Sichuan	1996	3	Yang et al. [33]
Chengdu, Sichuan	1999	63	Luo et al. [20]
Changsha	2008	1	Lei et al. [18]
Shanghai	2007–2009	12	Zhou et al. [38]
Shanghai	2009–2011	28	Zhou et al. [39]
Jiangsu	2012	1	Zhang et al. [37]

**Table 2:** Seroprevalence of *Toxocara* infection in children in the People’s Republic of China.

Provinces/cities	Year of sampling	No. of tested	No. of positive	Prevalence (%)	Method	Reference
Sichuan	1993	657	72	10.96	ELISA^a^	Luo et al. [19]
Chengdu, Sichuan	1999	557	64	11.49	ELISA	Luo et al. [20]
Weihai and Qingdao, Shandong	2011–2013	133	20	15.04	ELISA	Cong et al. [5]
Shandong and Jilin	2013–2014	1458	281	19.27	ELISA	Cong et al. [6]

a ELISA: enzyme-linked immunosorbent assay.

## Materials and methods

### Ethics statements

In the current study, all protocols were reviewed and approved by the Ethics Committee of Xinxiang Medical University (Ref. no. 2015018).

### Study site and sample collection

Blood samples were collected from 2451 primary school children in four cities of Henan province, as previously described [31]. The same sera were used in both studies.

### Antibodies to *Toxocara*

A commercial *Toxocara* IgG ELISA kit (Diagnostic Automation Inc., Woodland Hills, CA, USA) was used to detect anti-*Toxocara* IgG antibodies. Both negative and positive controls were provided in the kit and used in each test. Samples were considered positive on the basis of absorption that was no less than 0.3 OD units. Samples with inconclusive results were tested again. All the operations were performed according to the manufacturer’s instructions [5, 35]. The sensitivity and specificity of the ELISA were 87.5% and 93.3%, respectively.

### Statistical analysis

Statistical analysis was performed using SPSS 20 software for Windows (SPSS Inc., Chicago, IL, USA). Statistical analyses of *Toxocara* prevalence in different variables were performed using a *χ*^2^-test. A *p*-value of less than 0.05 was deemed statistically significant.

## Results

As shown in Table 3, the overall seroprevalence of *Toxocara* infection among primary school children in Henan province was 5.14%. *Toxocara* seroprevalence among the children living in Xinxiang, Zhengzhou, Zhumadian and Nanyang were 3.08%, 5.90%, 4.52%, and 7.08%, respectively. The prevalence of *Toxocara* antibodies varied significantly with the place of residence (*p* = 0.014).

**Table 3:** Seroprevalence of *Toxocara* infection in primary school children in Henan province, central China.

Variable	No. of tested	No. of positive	Prevalence (%)	95% CI	*χ*^2^	*p*-value
Region						
Xinxiang	585	18	3.08	1.68–4.48	10.637	0.014
Zhengzhou	696	41	5.90	4.14–7.64		
Zhumadian	619	28	4.52	2.89–6.16		
Nanyang	551	39	7.08	4.94–9.22		
Sex						
Male	1289	68	5.28	4.06–6.50	0.101	0.751
Female	1162	58	4.99	3.74–6.24		
Age (years)						
6–7	819	29	3.54	2.28–4.81	8.033	0.018
8–9	784	41	5.23	3.67–6.79		
10–11	848	56	6.60	4.93–8.28		
Residence area						
Urban	1127	46	4.08	2.93–5.24	4.799	0.028
Rural	1324	80	6.04	4.76–7.33		
Contact with cats						
No	1462	64	4.38	3.33–5.43	4.328	0.037
Yes	989	62	6.27	4.76–7.78		
Contact with dogs						
No	1328	51	3.84	2.81–4.87	10.051	0.002
Yes	1123	75	6.68	5.22–8.14		
Exposure to soil						
No	659	24	3.64	2.21–5.07	4.153	0.042
Yes	1792	102	5.69	4.62–6.76		
Hand washing before eating						
No	784	55	7.02	5.23–8.80	8.306	0.004
Yes	1667	71	4.26	3.29–5.23		
Total	2451	126	5.14	4.27–6.02		

There was no significant difference in the seroprevalence of *Toxocara* between boys and girls (5.28% vs. 4.99%, *χ*^2^ = 0.101, *p* = 0.751) (Table 3). The overall *Toxocara* seroprevalence increased with increasing age (Table 3). In comparison to groups of 6–7 year-olds (3.54%), and 8–9 year-olds (5.23%), the *Toxocara* seroprevalence amongst 10–11 year-olds was highest (6.60%).

The seroprevalence of *Toxocara* infection in children living in rural areas was significantly higher than those living in urban areas (*χ*^2^ = 4.799, *p* = 0.028), and the rate was significantly higher in children in contact with cats (*χ*^2^ = 4.328, *p* = 0.037) and dogs (*χ*^2^ = 10.051, *p* = 0.002). Moreover, the probability of infection with *Toxocara* was significantly increased in children in contact with soil compared to those with no contact (5.69% vs. 3.64%, *χ*^2^ = 4.153, *p* = 0.042). Children with the behavior of washing hands before eating exhibited lower seropositive rates for *Toxocara* than those without (4.26% vs. 7.02%, *χ*^2^ = 8.306, *p* = 0.004).

## Discussion

The overall seroprevalence for *Toxocara* infection was 5.14% in primary school children in Henan province. The seroprevalence obtained in this study was lower than that of other provinces in China such as Sichuan (10.96% and 11.49%) [19, 20], and Shandong and Jilin provinces (19.27%) [6]. Compared to other countries and regions, the total *Toxocara* seropositive rate in Henan province was also lower than 86.1% reported in children aged 7–17 years from Makoko, an urban slum community in Nigeria [12], 86.75% among students in primary schools from the capital area of the Republic of the Marshall Islands [10], 12.02% in the Amecameca and Chalco regions of México [7], 29.0% in Aragua state, Venezuela [22], and 10.0% in Serbia [11]. However, the seropositive rate was higher than that observed in Iran (1.39–3.8%) [13, 15, 16]. Several factors such as age of children, sample sizes, the specificity and sensitivity of the detection methods used, various climatic and geographical conditions, hygiene habits, and lifestyle of the population assessed could have contributed to the differences observed in seroprevalence rates.

The present study showed that geographical origin is a risk factor. The differences in seropositive rates among the different regions of Henan may be attributed to local food habits, climate condition, densities of population, and the number of stray dogs and cats. In this study, the seroprevalence value for boys was nearly equal to that of girls, indicating that the exposure levels of both sexes were very similar, and thus male or female sex was not a critical risk factor associated with *Toxocara* infection. This finding was consistent with other similar reports [7, 13].

It has been found in numerous studies that *Toxocara* seroprevalence is positively correlated with the age of children [12, 30]. In the current study, similarly, *Toxocara* seroprevalence also exhibited a positive correlation with the age of children, with a progressive and significant pattern. It has been hypothesized that this phenomenon results from increasing years of exposure as the child grows, and a generally highly contaminated environment.

The present study found that children with a history of contact with dogs or cats showed a higher tendency to acquire *Toxocara* infection than those who do not. A number of studies showed the presence of embryonated *T. canis* and *T. cati* eggs on the hair of dogs and cats, respectively, indicating that direct contact with dogs or cats may be a potential route of infection [1, 8, 23]. If children touch dogs and cats frequently, the possibility of accidentally ingesting embryonated *T. canis* or *T. cati* eggs from the hair increases, which results in a higher risk of infection. The seropositive rate of *Toxocara* in children living in rural areas was significantly higher than that in urban areas, which was consistent with other reports [16, 29]. The explanation for this observation is that the higher number of stray and domesticated dogs and cats in rural areas may have increased the degree of environmental contamination with *Toxocara* eggs. Poor hygiene habits in rural areas can also lead to a higher incidence of toxocariasis.

Soil contamination with *Toxocara* eggs is considered to be the main source of human infections [2, 25, 26]. The present study also revealed that exposure to soil was related to *Toxocara* infection in primary school children, indicating that soil contamination by dog and cat feces is widespread in these areas. In the future, more research is needed to evaluate the prevalence of *Toxocara* eggs in the soil of local parks and primary schools.

Additionally, hand washing before eating has been verified as a protective factor related to *Toxocara* seroprevalence in this study. This finding is in line with other similar surveys [3, 17].

## Conclusion

In conclusion, the present study revealed for the first time that *Toxocara* infection in primary school children is relatively common in Henan province, China. Prevention approaches including cleaning hands after contact with soil, cats or dogs and before eating, reducing soil contamination by dog or cat feces in public areas, and treating dogs and cats with anthelmintics to reduce *Toxocara* burdens, can be beneficial to minimize exposure to *Toxocara* spp.

## Conflict of interest statement

We declare that we have no conflict of interest.
